# Gamma tACS over the prefrontal and parietal cortices enhances episodic memory performance

**DOI:** 10.3389/fnhum.2026.1775435

**Published:** 2026-03-02

**Authors:** Kenta Honma, Tomonori Nomura

**Affiliations:** Faculty of Rehabilitation, Niigata University of Health and Welfare, Niigata, Japan

**Keywords:** transcranial alternating current stimulation (tACS), gamma oscillations, episodic memory, memory consolidation, prefrontal cortex, posterior parietal cortex, cognitive enhancement

## Abstract

**Background:**

Episodic memory is a critical component of daily functioning and is vulnerable to aging and neurological disorders. Gamma-frequency transcranial alternating current stimulation (tACS) has been proposed as a non-invasive approach to modulate memory-related neural activity.

**Objective:**

This randomized, sham-controlled study examined whether gamma-frequency tACS applied to the left prefrontal cortex (PFC) and posterior parietal cortex (PPC) during encoding and retrieval is associated with differences in episodic memory performance in healthy young adults.

**Methods:**

A total of 51 right-handed adults with no underlying health issues (mean age = 20.9 ± 1.0 years) were randomly assigned to one of three groups: two-site stimulation over the left PFC and PPC (PFC–PPC, *n* = 17), single-site stimulation over the left PFC (PFC, *n* = 17), or sham stimulation (*n* = 17). Participants completed a verbal recognition task across three sessions (Days 1, 2, and 7). On Days 1 (learning phases) and 2 (recognition phases), 60 Hz tACS (1.5 mA) was delivered. The primary outcome was the discrimination index (d-prime) on Day 7. Accuracy and d-prime were analyzed using two-way repeated-measures analysis of variance and effect sizes (Hedges' g).

**Results:**

Significant effects of time and time-by-group interactions were observed for accuracy and d-prime. The PFC–PPC group showed higher d-prime scores than the sham group on Days 2 and 7, with medium-to-large effect sizes. Single-site PFC stimulation was also associated with numerically higher d-prime scores relative to sham on Day 7. Although effect sizes were larger in the PFC–PPC group than in the PFC group, direct comparisons between the two active stimulation conditions did not yield statistically robust differences.

**Conclusions:**

These findings provide preliminary behavioral evidence that gamma-frequency tACS delivered to memory-related cortical regions is associated with differences in episodic memory discrimination at delayed time points. However, the results are based solely on behavioral measures, and replication with larger and more diverse samples, as well as studies incorporating neurophysiological recordings, will be necessary to clarify the underlying mechanisms and robustness of these effects.

## 1 Introduction

Episodic memory, which is the ability to encode and retrieve personally experienced events with contextual detail, is fundamental to daily functioning and the continuity of subjective experiences (Tulving, 1984, 2002). This memory system is also highly vulnerable to aging and neuropsychiatric conditions, such as mild cognitive impairment and dementia (Chatzikostopoulos et al., 2022). Consequently, there is an increasing interest in neuromodulatory interventions that may enhance episodic memory or protect against decline. Among these, transcranial alternating current stimulation (tACS) has gained prominence as a method for entraining brain oscillations in a frequency-specific manner to modulate the dynamics of large-scale neural networks (Lang et al., 2019; Shtoots et al., 2024).

Accumulating evidence highlights the importance of gamma-band oscillations in episodic memory. Gamma synchronization has been linked to the binding of item-context information (Nyhus and Curran, 2010), coordination of distributed neural assemblies (Griffiths and Jensen, 2023), and reactivation of stored representations during retrieval (Osipova et al., 2006). The prefrontal cortex (PFC), a key hub within the episodic memory network, supports top–down strategic control and successful encoding operations (Javadi et al., 2017). Several studies have demonstrated that applying gamma-frequency tACS to the PFC during encoding improves episodic memory performance in recognition and associative learning tasks (Nomura et al., 2019; Grover et al., 2022), suggesting that externally driven gamma activity strengthens memory trace formation (Mencarelli et al., 2022). Furthermore, a recent review reported that gamma tACS has the potential to improve memory performance in individuals with mild cognitive impairment or Alzheimer's disease (Nissim et al., 2023).

Beyond the PFC, recent studies have indicated that the posterior parietal cortex (PPC) plays a central role in episodic memory. PPC are involved in memory-guided attention, evidence accumulation during recognition decisions, and the reinstatement of previously acquired information (Vilberg and Rugg, 2008; Fischer et al., 2021). Gamma-band stimulation of the parietal areas has been reported to enhance long-term memory performance (Sun et al., 2025), potentially via interactions with hippocampal–cortical consolidation processes (Benussi et al., 2022). However, most prior stimulation studies have examined either the PFC or PPC in isolation, and the effects have typically been measured immediately or shortly after stimulation (Biel et al., 2022; Hu et al., 2022; Xiao et al., 2023). Moreover, a previous study applying gamma tACS to prefrontal and parietal regions observed alterations in memory confidence bias, whereas effects on objective behavioral performance were less consistent (Wynn et al., 2025). Consequently, it remains unknown whether stimulating multiple nodes in the memory network yields additive or synergistic benefits and whether such improvements extend to longer retention intervals relevant to real-world memory functions.

To address these gaps, we conducted a randomized sham-controlled study to test whether gamma-frequency tACS applied over the left PFC and PPC enhances episodic memory performance when administered during encoding and retrieval. The participants completed a verbal recognition task at three time points (Days 1, 2, and 7) and were randomly assigned to one of three groups: (i) two-site stimulation over the left PFC and PPC, (ii) single-site stimulation over the left PFC, and (iii) sham stimulation. The primary outcome measure was d-prime discrimination accuracy on Day 7, indexing long-term episodic memory retention.

We hypothesized that two-site gamma tACS would yield the greatest improvement in delayed memory performance, followed by single-site PFC stimulation, relative to sham stimulation. We further predicted that these effects would persist across sessions, particularly at the Day 7 assessment.

## 2 Methods

### 2.1 Participants

This study included 51 healthy adults (20 males and 31 females). The mean age ± standard deviation (SD) was 20.9 ± 1.0 years, and the mean years of education ± SD was 15.0 ± 1.1 years.

The sample size was determined using G^*^Power 3.1 software (Kang, 2021), assuming the expected effect size (Cohen's *f* = 0.25) and the statistical power (1–β) was 0.80, based on the planned repeated-measurement statistical analysis. All participants were right-handed, with Edinburgh Handedness Inventory scores ranging from 0.8 to 1.0 (Oldfield, 1971). Participants were randomly assigned to one of three groups: the PFC–PPC stimulation group (*n* = 17, 11 women, SD mean age, 20.9 ± 0.7), the PFC stimulation group (*n* = 17, 11 women, SD mean age, 21.0 ± 1.0) or the sham stimulation group (*n* = 17 women, SD mean age, 21.0 ± 1.3) based on Helsinki Declaration. This study was conducted in accordance with the Declaration of Helsinki, and the experimental protocol was approved by the Ethics Committee of Niigata University of Health and Welfare (approval number: 19503-250219). The inclusion criteria were as follows: participants had to be native Japanese speakers, non-smokers, right-handed, with a normal or normal-looking appearance, and without a history of neurological trauma or mental illness. The exclusion criteria included a history of head injuries, presence of metal implants, epileptic seizures, and current use of psychotropic medications. Participants with scores above 45 on the self-evaluative depression scale (SDS) were excluded (Zung, 1965).

To verify general memory function at a fundamental level, participants completed the Attention/Concentration Index of the Wechsler Memory Scale-Revised (WMS-R) in Japan prior to registration (Koike and Sugishita, 2011). In addition, to evaluate baseline episodic memory performance, an episodic memory task from a previous study was used (Nomura et al., 2019). Participants were instructed to learn 50 Japanese words in a preliminary learning phase conducted separately from the main experiment, with each word presented for 1 s on the screen. Following the learning phase, the participants completed a recognition task consisting of 100 words, including 50 previously learned (“old”) words and 50 new words. Memory performance was evaluated using d-prime and accuracy (Table 1).

**Table 1:** Baseline characteristics of the participants: mean (SD).

**Variable**	**PFC–PPC (*n* = 17)**	**PFC (*n* = 17)**	**Sham (*n* = 17)**	***p*-value**
Age (years)	20.9 (0.7)	21.0 (1.0)	21.0 (1.3)	0.91^b^
Sex (female)	11	11	9	0.72^c^
Education (years)	14.8 (0.7)	15.1 (1.2)	15.1 (1.3)	0.42^b^
Edinburgh handedness inventory (0–1)	0.99 (0.03)	0.97 (0.06)	0.99 (0.06)	0.42^b^
Self-rating depression scale	33.9 (2.5)	36.8 (6.7)	35.3 (7.9)	0.42^b^
WMS-R Attention/Concentration score	96.4 (12.9)	98.2 (12.9)	99.0 (10.7)	0.70^a^
**Episode memory task (50 words)**				
d-prime	2.03 (0.7)	1.84 (0.5)	1.72 (0.6)	0.34^a^
Accuracy	0.81 (0.09)	0.80 (0.07)	0.79 (0.1)	0.75^a^

^a^One-way Analysis of Variance.

^b^Kruskal–Wallis test.

^c^Chi-squared test.

### 2.2 Procedures

This study employed a single-blind, sham-controlled, three-arm, repeated-measurement design. The experiment was conducted over three distinct sessions on Days 1, 2, and 7.

**Figure 1:**
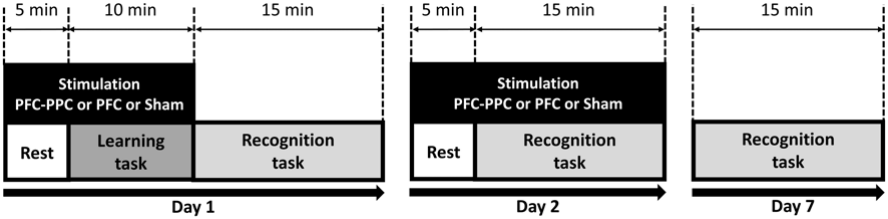
Study design for the episodic memory task. Participants conducted a learning task on Day 1, and memory recognition tasks were administered on Days 1, 2, and 7. Participants received stimulation (PFC–PPC or PFC or sham) on Days 1 and 2. On Day 7, only memory recognition tasks were performed.

On the first day, the participants completed a learning task to encode “old” words for the episodic memory task. All participants received tACS (PFC–PPC, PFC, or sham) during the learning task on Day 1 and during the recognition task on Day 2; the order of stimulation was fixed and not counterbalanced. Recognition performance was assessed on Days 1, 2, and 7 (Figure 1).

Participants' recognition performance was assessed using a two-alternative forced-choice (2 AFC) new/old recognition task. Participants were tasked with the evaluation of each word presented on its novelty, categorizing it as either “old” (previously learned) or “new.”

### 2.3 Episodic memory task

The episodic memory task was performed as described by Nomura et al. (2019). A database obtained from elementary schools in Japan was used to select 400 Japanese nouns. The nouns were kanji (73.8%) and katakana (26.2%).

Kanji words were subjected to a control procedure that considered the number of letters and ranged from one to three characters, with a mean (SD) length of 1.86 (0.04) characters. Katakana words were also controlled for length and ranged from one to four characters, with a mean (SD) length of 3.39 (0.08) characters. The selection of words was met with a high degree of familiarity by the participants, who reported comprehensive knowledge of the entire set.

**Figure 2:**
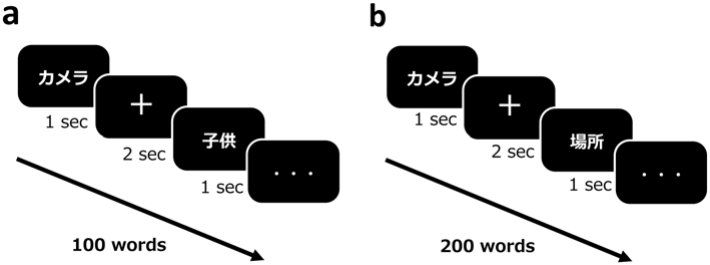
Overview of the episodic memory task. **(a)** Participants learned 100 well-known, easy words (“old words”) on Day 1. Each word was displayed for 1 s, and a cross-symbol was displayed for 2 s between words. **(b)** The recognition task presented a total of 200 words, a combination of 100 old words and 100 new words. Each word was displayed for 1 s, and a cross-symbol was displayed for 2 s between words. The recognition task was applied as a two-alternative forced-choice task (new or old words).

Participants were required to learn 100 of these familiar words (referred to as “old words”) during the learning task. Each word was presented for a duration of 1 s, with a fixation cross presented for 2 s between the words (Figure 2a).

The recognition task comprised a total of 200 words, including 100 previously learned (“old”) words from the learning task and 100 new words. Across all sessions (Days 1, 2, and 7), the same old words were used in each session, whereas a different set of new words was used in each session. During the recognition phase, each word was presented for 1 s, followed by a fixation cross displayed for 2 s between words (Figure 2b).

Participants were instructed to hold a response button in each hand and press the button on the right when an old word was shown, and the button on the left when a new word was shown. Participants were requested to respond with the utmost accuracy and expediency.

All task sessions were conducted in the same room across the three testing days. The experiment was administered on a personal computer equipped with a 24-inch monitor. The memory task was presented using the Multi-Trigger System (Medical Type System, Tokyo, Japan) in white 150-point Gothic font on a black background.

### 2.4 Transcranial alternating current stimulation (tACS)

tACS was performed using the DC Stimulator PLUS (NeuroConn GmbH, Ilmenau, Germany).

Electrical stimulation was applied via two 5 × 7 cm rubber electrodes with saline-soaked surface sponges. To reduce contact impedance, a conductive gel was applied beneath each electrode prior to mounting (Manenti et al., 2013; Sandrini et al., 2014, 2016; Nomura et al., 2019).

Electrode placement was conducted in accordance with the international 10–20 system (Polania et al., 2012; Nomura et al., 2019; Biel et al., 2022). For the present study, electrodes were placed at F3 (left PFC) and P3 (left PPC) in the PFC–PPC and sham groups, with an additional inactive electrode placed over the right deltoid muscle. In the PFC group, electrodes were positioned at F3 and the right deltoid muscle, whereas an inactive electrode was placed at P3. To maintain blinding, electrodes were placed at all three locations (F3, P3, and the right deltoid) for all participants, and the electrode cables were concealed.

For active tACS, a sinusoidal current of 1.5 mA (current density: 0.06 mA/cm^2^) was delivered at 60 Hz (Nomura et al., 2019; Sun et al., 2025). The stimulator employed a fade-in/fade-out mode, whereby the current was increased over a 10-s period at the commencement of stimulation and subsequently decreased over a 10-s period at the cessation of stimulation. In the sham group, the same ramp procedure was applied, but stimulation was discontinued after 10 s.

It has been hypothesized that alterations in cortical excitability transpire approximately 5 min after the initiation of stimulation (Nitsche and Paulus, 2000). Moreover, the application of 60 Hz tACS during the encoding and retrieval phases has been demonstrated to enhance memory performance (Javadi et al., 2017). Accordingly, on Day 1, electrical stimulation was initiated 5 min before the onset of the learning task and continued for 10 min during task performance, resulting in a total stimulation duration of 15 min. On Day 2, stimulation was similarly initiated 5 min before the onset of the recognition task and continued for 15 min during task performance, yielding a total stimulation duration of 20 min (Figure 1). All tACS procedures adhered to established safety guidelines (Antal et al., 2017).

**Figure 3:**
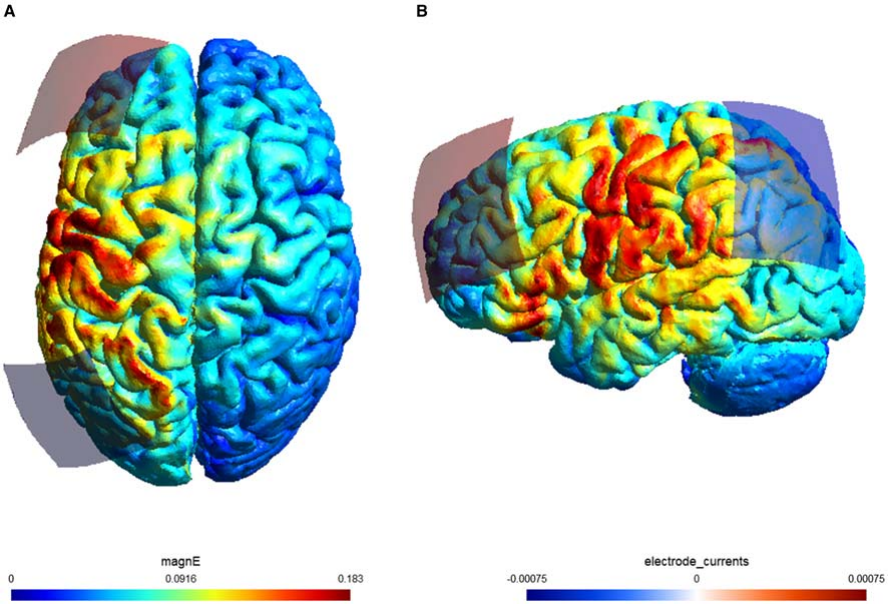
Electric field distribution simulated with SimNIBS for the PFC–PPC stimulation montage used in the experiment (electrodes positioned over F3 and P3). **(A)** Superior (top) view of the electric field magnitude (|E|). **(B)** Left lateral view of the same simulation. Simulations were performed using SimNIBS 4.0 with a standard head model and a 5 × 7 cm rectangular electrode under 1.5 mA, 60-Hz alternating current stimulation. Warmer colors indicate stronger electric field magnitudes.

In the PFC–PPC group, tACS was delivered using a two-electrode montage between F3 and P3. In the PFC group, stimulation was delivered between F3 and an extracephalic reference electrode placed over the right deltoid muscle. To supplement the description of the stimulation configuration, finite element modeling was conducted using SimNIBS 4.0 to illustrate the spatial distribution of the induced electric field for the PFC–PPC stimulation condition. Electric field simulations were performed using a standard head template with electrodes positioned over the left PFC (F3) and left PPC (P3). As shown in Figure 3, the simulated electric field was not confined exclusively to the nominal electrode locations but extended to adjacent cortical regions, including portions of the precentral and postcentral gyri, illustrating the distributed nature of current flow in tACS.

### 2.5 Statistical analysis

The primary outcome of this study was d-prime on Day 7. The secondary outcomes were d-prime on Days 1 and 2 and accuracy on Days 1, 2, and 7.

To facilitate a comparison of baseline characteristics across groups, descriptive statistics were calculated, and Shapiro–Wilk tests were used to assess normality. A range of between-group differences was examined, including age, years of education, Edinburgh Handedness Index, Self-Rating Depression Scale score, WMS-R Attention/Concentration score, baseline episodic memory task accuracy, and d-prime. Following the confirmation of normality, one-way analysis of variance (ANOVA) was employed; in instances where normality was violated, the Kruskal–Wallis test was used. The chi-square test was used to analyze sex differences among the study groups.

Accuracy and d-prime were analyzed as measures of episodic memory performance. In the 2 AFC recognition task, participants judged each word as either old or new. The discrimination index (d-prime), derived from signal detection theory (Snodgrass and Corwin, 1988), reflects the ability to distinguish targets from distractors while accounting for false alarms (Haatveit et al., 2010). d-prime was calculated as the difference between z-transformed hit rates and false alarm rates.

To examine the effect of tACS on memory retention, two-factor repeated-measures ANOVAs were conducted for accuracy and d-primes. The between-subjects factor was the stimulation condition (PFC–PPC, PFC, sham), and the within-subjects factor was time (Days 1, 2, and 7). In instances where substantial main effects or interactions were identified, *post-hoc* pairwise comparisons were conducted with Bonferroni adjustment for multiple comparisons.

Day 7 d-prime was pre-specified as the primary outcome measure. All other analyses, including accuracy and earlier time points, were considered secondary and exploratory. In addition to *p*-values, Hedges' g was calculated to estimate between-group effect sizes for accuracy and d-prime, along with their 95% confidence intervals.

All statistical analyses were performed using the IBM SPSS Statistics, Version 31 (IBM Corp., Armonk, NY, USA). Statistical significance was set at *p* < 0.05 (two-tailed).

## 3 Results

### 3.1 Baseline characteristics

The demographic and neuropsychological characteristics of the three groups at the initial point of measurement are summarized in Table 1. The study revealed no statistically significant differences among the PFC–PPC, PFC, and sham groups with respect to age, years of education, Edinburgh Handedness Index, Self-Rating Depression Scale scores, or WMS-R Attention/Concentration index (all *p* > 0.10). Furthermore, no significant group differences were observed in the baseline episodic memory task performance (i.e., accuracy or d-prime) obtained in the separate pre-experimental sessions (all *p* > 0.10). These findings suggest that the three groups were comparable at baseline in terms of demographic variables, mood, general memory function, and episodic memory performance.

### 3.2 Adverse effects

No serious adverse events were observed. In the PFC–PPC group, seven participants reported mild itching or tingling sensations under the electrodes, whereas two participants in the PFC group reported similar sensations. All nine participants indicated that these sensations occurred only at the onset of stimulation and gradually subsided over time. These sensations did not interfere with task performance, and none of the participants reported pain or any other discomfort. The frequency and intensity of the neurosensory sensations did not differ markedly between the active and sham groups, and no clinically relevant neurosensory side effects were observed.

### 3.3 Episodic memory performance

**Figure 4:**
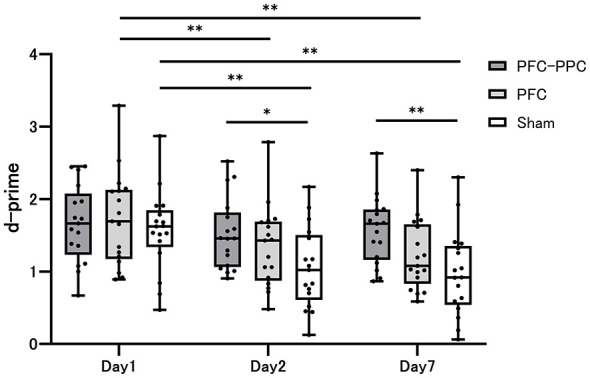
The d-prime at 3 time points for the PFC–PPC group, PFC group, and Sham group. A two-way repeated-measures ANOVA of d-prime revealed significant main effects of time and the interaction of intervention and time. A *post-hoc* analysis revealed significant differences between the PFC–PPC group and the sham group on Days 2 and 7. *p < 0.05, **p < 0.01.

**Figure 5:**
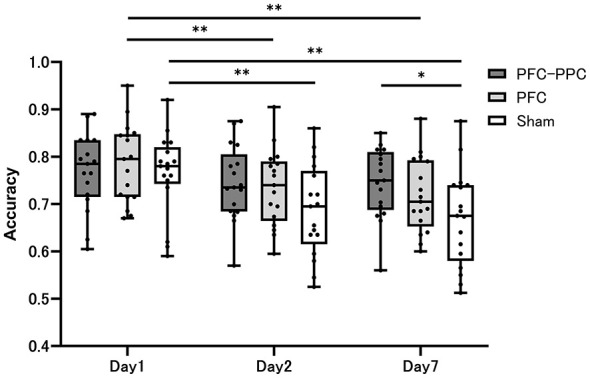
The Accuracy at 3 time points for the PFC–PPC group, PFC group, and Sham group. A two-way repeated-measures ANOVA of accuracy revealed significant main effects of time and the interaction of intervention and time. A *post-hoc* analysis revealed significant differences between the PFC–PPC group and the sham group on Day 7. *p < 0.05, **p < 0.01.

Descriptive statistics for d-prime and accuracy in the episodic memory task at each time point (Days 1, 2, and 7) for each group are presented in Table 2. Figures 4, 5 illustrate the time courses of d-prime and accuracy, respectively, across the three groups. Effect sizes (Hedges' g) for all between-group comparisons at each time point are reported in Table 3.

**Table 2:** The episodic memory task for each group and session: mean (SD).

**Outcome measure**	**Group**	**Day1**	**Day2**	**Day7**
d-prime	PFC–PPC	1.66 (0.53)	1.51 (0.49)	1.57 (0.46)
	PFC	1.72 (0.65)	1.35 (0.56)	1.22 (0.48)
	Sham	1.57 (0.56)	1.09 (0.54)	0.96 (0.58)
Accuracy	PFC–PPC	0.77 (0.08)	0.74 (0.08)	0.75 (0.07)
	PFC	0.79 (0.08)	0.73 (0.08)	0.71 (0.08)
	Sham	0.77 (0.08)	0.70 (0.09)	0.69 (0.10)

**Table 3:** Effect sizes in Hedges‘ *g* values.

**Group comparison**	**Group differences**	**Day1, Hedges' *g* [95%Cl]**	**Day2, Hedges' *g* [95%Cl]**	**Day7, Hedges' *g* [95%Cl]**
d-prime	PFC–PPC vs. PFC	−0.10 [−0.77, 0.57]	0.32 [−0.36, 0.10]	0.72 [−0.02, 1.41]
	PFC–PPC vs. Sham	0.16 [−0.52, 0.83]	0.81 [0.11, 1.51]	1.13 [0.40, 1.86]
	PFC vs. Sham	0.24 [−0.44, 0.91]	0.46 [−0.22, 1.14]	0.48 [−0.21, 1.16]
Accuracy	PFC–PPC vs. PFC	−0.20 [−0.88, 0.47]	0.11 [−0.56, 0.78]	0.35 [−0.33, 1.03]
	PFC–PPC vs. Sham	0.06 [−0.61, 0.73]	0.63 [−0.06, 1.32]	0.83 [0.12, 1.53]
	PFC vs. Sham	0.25 [−0.42, 0.93]	0.53 [−0.16, 1.21]	0.52 [0.12, 1.53]

### 3.4 d-prime (primary outcome)

For the primary outcome, d-prime, the repeated-measures ANOVA revealed a significant main effect of time [*F*_(2, 96)_ = 33.851, *p* < 0.001, η^2^ = 0.414] and a significant group × time interaction [*F*_(4, 96)_ = 4.627, *p* = 0.002, η^2^ = 0.162]. *Post-hoc* analyses indicated that d-prime significantly declined over time in the PFC and sham groups (Figure 4). In between-group comparisons at each time point, the PFC–PPC group demonstrated significantly higher d-prime scores than the sham group on Day 2 (*p* = 0.015) and Day 7 (*p* < 0.001) (Figure 4). The corresponding effect sizes ranged from medium to large. On Day 7, the PFC group showed a numerically higher d-prime compared with the sham group, with a moderate effect size; however, the 95% confidence interval for Hedges' g included zero, indicating that this difference was not statistically robust. Direct comparisons between the two active stimulation groups (PFC–PPC vs. PFC) did not reveal statistically significant differences in d-prime at any time point (Table 3).

### 3.5 Accuracy

For accuracy, the two-way repeated-measures ANOVA revealed a significant main effect of time [*F*_(2, 96)_ = 33.967, *p* < 0.001, η^2^ = 0.414] and a significant group × time interaction [*F*_(4, 96)_ = 3.50, *p* = 0.01, η^2^ = 0.172]. *Post-hoc* analyses indicated that accuracy significantly declined over time in the PFC and sham groups (Figure 5). In between-group comparisons at each time point, the PFC–PPC group demonstrated significantly higher accuracy than the sham group on Day 7 (*p* = 0.013) (Figure 5). The corresponding effect size ranged from small to moderate. The PFC group showed numerically higher accuracy compared with the sham group at the delayed time point; however, this difference did not reach statistical significance after correction, and the confidence interval of the effect size included zero, indicating that the effect was not statistically robust. Direct comparisons between the two active stimulation groups (PFC–PPC vs. PFC) did not reveal statistically significant differences in accuracy at any session (Table 3).

To further illustrate individual variability in memory performance across sessions, individual trajectories of d-prime and accuracy for all participants, together with group mean trends, are shown in Supplementary Figures S1, S2, respectively.

## 4 Discussion

The key finding of the present study was that gamma-frequency tACS was associated with a reduced decline in memory performance over time, suggesting a potential role in supporting longer-term memory retention.

This randomized, sham-controlled study examined the effects of gamma-frequency tACS targeting memory-relevant cortical regions on episodic memory performance in healthy young adults. Participants who received two-site stimulation over the left PFC and PPC showed relatively higher discrimination performance at delayed time points compared with those who received sham stimulation. Single-site PFC stimulation was also associated with numerically higher delayed performance relative to sham, although differences between the two active stimulation conditions were not statistically significant. Taken together, these findings provide preliminary evidence that gamma-band neuromodulation may help support the preservation and longer-term retention of episodic information.

These results are broadly consistent with previous studies reporting that gamma-frequency tACS applied to the PFC is associated with changes in associative and recognition memory performance (Nomura et al., 2019; Grover et al., 2022). The present findings extend this literature by suggesting that stimulation delivered during encoding and retrieval may be linked to memory effects that persist for several days after stimulation. This interpretation is compatible with theoretical accounts proposing that gamma-band synchronization contributes to memory processing by supporting long-range cortical communication and top–down control (Javadi et al., 2017; Roux et al., 2022). Rather than demonstrating direct enhancement of memory, the present results may reflect modulation of processes related to memory stability or retention over time.

The present work also aligns with evidence indicating that the PPC contributes to episodic memory through processes such as attentional guidance, reinstatement of previously encoded information, and early cortical consolidation (Vilberg and Rugg, 2008; Brodt et al., 2016). Previous studies reporting memory-related effects following gamma-frequency stimulation of parietal regions (Sun et al., 2025) suggest that the PPC may be involved in memory-related modulation. In the present study, numerically larger effects were observed in the two-site stimulation condition compared with the single-site condition. However, these differences were not statistically significant and should therefore be interpreted with caution. The current results do not provide evidence for additive or synergistic effects of multi-site stimulation, but rather indicate that both stimulation approaches were associated with comparable behavioral outcomes within the limits of the present design. It should also be noted that the electric field simulation conducted in the present study indicated that the induced electric field was not confined exclusively to the nominal electrode locations over the PFC and PPC. Consistent with prior modeling studies of transcranial electrical stimulation, the simulated field extended to adjacent cortical regions, including the precentral and postcentral gyri, reflecting the inherently diffuse nature of current distribution in tACS (Antal and Paulus, 2013; Herrmann et al., 2013; Saturnino et al., 2019). Accordingly, the behavioral effects observed in the present study cannot be attributed solely to focal modulation of the PFC or PPC. Rather, it is possible that stimulation influenced a broader set of cortical regions functionally connected to memory-related processes. Although the electric field strength in medial temporal structures was limited in the present simulation, indirect modulation of memory performance via distributed cortical interactions cannot be excluded. Previous work has emphasized that episodic memory depends on coordinated activity across multiple cortical and subcortical regions, rather than isolated stimulation of a single cortical site (Vilberg and Rugg, 2008; Brodt et al., 2016). Importantly, while the stimulation montage was designed to target the PFC and PPC, the present findings should be interpreted as reflecting modulation of memory-related processes at a systems level, rather than selective stimulation of discrete cortical loci. Given that no neurophysiological measures were collected, the present study cannot directly determine which specific regions or pathways were responsible for the observed behavioral effects.

The absence of a statistically significant difference between the two-site and single-site stimulation conditions warrants cautious interpretation. The present study was not specifically powered to detect small-to-moderate differences between the two active stimulation protocols, and the overlapping confidence intervals indicate substantial uncertainty in the estimated effect sizes. Taken together, the findings suggest that gamma-frequency tACS applied over the PFC or a broader frontoparietal region may be associated with differences in episodic memory performance relative to sham stimulation. However, the current data do not provide clear evidence supporting the superiority of multi-site stimulation. Future studies with larger sample sizes and the inclusion of neurophysiological measures will be required to more precisely evaluate potential differences between stimulation approaches.

Within the broader non-invasive brain stimulation literature, repetitive transcranial magnetic stimulation (rTMS) has also been investigated as a method for cognitive enhancement, including effects on memory-related functions. Recent studies applying rTMS over the dorsolateral prefrontal cortex have reported cognitive and memory-related effects in healthy adults, situating rTMS as a complementary approach to tACS in the modulation of higher-order cognitive processes (Ebrahimzadeh et al., 2025; Asgarinejad et al., 2024). Compared with rTMS, which induces relatively focal and transient perturbations of cortical excitability, tACS is generally interpreted as modulating ongoing neural activity in a frequency-specific manner, potentially aligning more closely with hypotheses regarding rhythmic coordination during episodic memory processing (Asgarinejad et al., 2024). In addition, rTMS studies—particularly in clinical populations—have emphasized substantial inter-individual variability in responsiveness and have increasingly employed computational and machine-learning approaches to predict treatment outcomes, highlighting the importance of individual differences when interpreting stimulation effects (Ebrahimzadeh et al., 2021, 2023, 2024). Taken together, comparisons with rTMS studies help situate the present tACS findings within the broader landscape of non-invasive brain stimulation, suggesting that different stimulation modalities may influence episodic memory through partially overlapping but not identical mechanisms.

These results may be relevant for the development of non-pharmacological interventions targeting populations vulnerable to memory decline, such as older adults or individuals with mild cognitive impairment. The observation that brief sessions of stimulation produced improvements that were detectable several days later supports the idea that tACS may influence consolidation processes rather than only transient cognitive states (Ketz et al., 2018). However, given that the present findings were observed in healthy young adults, the extent to which they can be generalized to aging or clinical populations remains unknown.

This study has some limitations that should be acknowledged. First, the present findings are based exclusively on behavioral measures, and no neurophysiological recordings (e.g., electroencephalography or functional magnetic resonance imaging) were collected. As a result, the neural mechanisms potentially underlying the observed effects of gamma-frequency tACS cannot be directly assessed and remain speculative. Second, because stimulation was applied during encoding and retrieval phases, it is not possible to disentangle the relative contributions of stimulation at each stage to the observed memory effects. Third, although stimulation electrodes were placed over the left PFC (F3) and PPC (P3), electric field simulations indicated that the induced field was distributed across a broader set of cortical regions, including adjacent sensorimotor areas such as the precentral and postcentral gyri. Therefore, the observed behavioral effects cannot be attributed exclusively to modulation of the targeted PFC or PPC regions, and contributions from neighboring cortical areas cannot be excluded. Fourth, the sample size was modest, which may have limited statistical power to detect differences between the two active stimulation conditions. Fifth, substantial inter-individual variability in responsiveness to tACS was observed, as illustrated in Supplementary Figures 1, 2. Although the present study was not designed to formally classify responders and non-responders, these findings highlight the importance of considering individual differences in future, adequately powered studies. Finally, only a single stimulation frequency and montage were examined, leaving the frequency specificity and optimal stimulation parameters unclear. In addition, repeated exposure to the same task materials across sessions may have contributed to retesting effects, and the effectiveness of blinding was not formally assessed. The study was not preregistered, and analyses beyond the prespecified primary outcome should be interpreted as exploratory.

Future studies should extend this work by examining the effects of gamma-frequency tACS in aging and clinical populations, where memory decline is more pronounced and clinical relevance may be greater. In addition, combining tACS with electrophysiological or neuroimaging measures will be essential to directly assess the neural processes underlying behavioral effects and to clarify the relative contributions of stimulation during encoding and retrieval. Adequately powered designs will also be necessary to more conclusively compare single-site and multi-site stimulation approaches and to optimize stimulation frequency and montage. Finally, the use of individualized targeting and state-dependent stimulation protocols may further refine our understanding of how gamma-band tACS influences memory-related processes at the network level.

## 5 Conclusion

In summary, the present study provides preliminary behavioral evidence that gamma-frequency tACS applied during encoding and retrieval is associated with differences in episodic memory discrimination in healthy young adults. PFC-only and frontoparietal stimulation were associated with relatively better delayed performance compared with sham stimulation, with numerically larger effects observed for the two-site protocol. However, these effects were assessed using behavioral measures only, and differences between the two active stimulation conditions were not statistically robust. Accordingly, although the present findings suggest that gamma-band tACS may support the preservation of episodic memory performance over time, further studies incorporating neurophysiological measures and larger samples are required to clarify the underlying mechanisms and to determine the optimal stimulation parameters.

## Data Availability

The raw data supporting the conclusions of this article will be made available by the authors, without undue reservation.
