# Supplementary material for: Gamma tACS over the prefrontal and parietal cortices enhances episodic memory performance
Source: Front Hum Neurosci. 2026 Mar 2;20:1775435. doi: 10.3389/fnhum.2026.1775435 (PMC12989554; doi:10.3389/fnhum.2026.1775435)
Supplement: Supplementary file 1 [file Data_Sheet_1.pdf]

Supplementary Figure S1. Individual and group-level changes in d-prime across testing sessions. Thin lines represent individual participants' d-prime scores measured on Days 1, 2, and 7 within each stimulation group (PFC–PPC, PFC, and sham). Thick lines indicate the group mean values across time points for each condition.

Supplementary Figure S2. Individual and group-level changes in accuracy across testing sessions. Thin lines represent individual participants' accuracy scores measured on Days 1, 2, and 7 within each stimulation group (PFC–PPC, PFC, and sham). Thick lines indicate the group mean values across time points for each condition.
