# Supplementary figures and images for: Gamma tACS over the prefrontal and parietal cortices enhances episodic memory performance
Source: Front Hum Neurosci. 2026 Mar 2;20:1775435. doi: 10.3389/fnhum.2026.1775435 (PMC12989554; doi:10.3389/fnhum.2026.1775435)

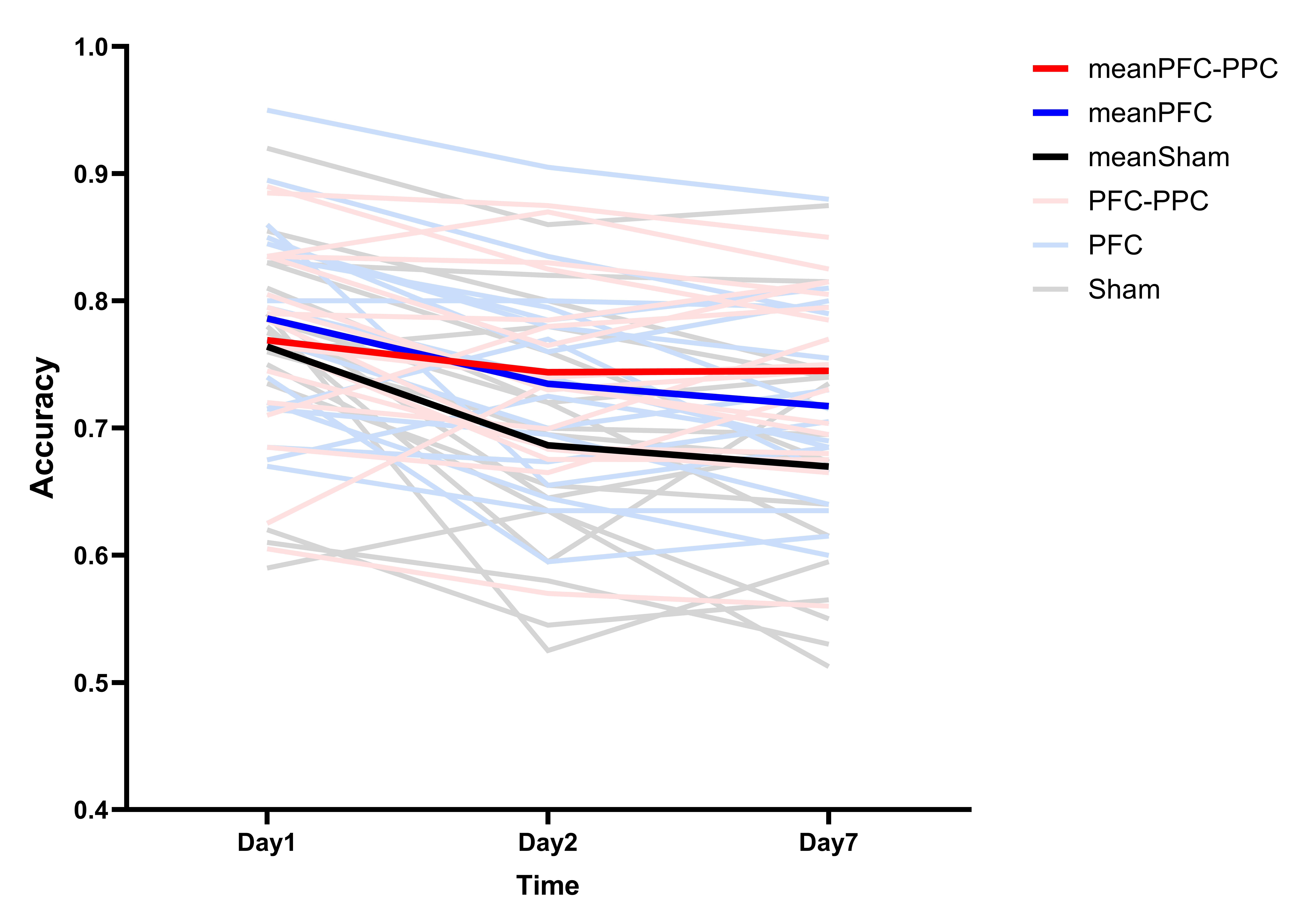

Supplement: Supplementary file 3 [file Image_2.tif]
